# In silico mutagenesis-based designing of oncogenic SHP2 peptide to inhibit cancer progression

**DOI:** 10.1038/s41598-023-37020-4

**Published:** 2023-06-21

**Authors:** Muhammad Shahab, Shahin Shah Khan, Maryam Zulfat, Yousef A. Bin Jardan, Amare Bitew Mekonnen, Mohammed Bourhia, Guojun Zheng

**Affiliations:** 1 State Key Laboratories of Chemical Resources Engineering, Beijing University of Chemical Technology, Beijing, 100029 China; 2 College of Life Science and Technology, Beijing University of Chemical Technology, Beijing, 100029 China; 3 Department of Chemistry, Computational Medicinal Chemistry Laboratory, UCSS, Abdul Wali Khan University, Mardan, Pakistan; 4 Department of Pharmaceutics, College of Pharmacy, King Saud University, Riyadh, Saudi Arabia; 5 Department of Biology, Bahir Dar University, P.O. Box 79, Bahir Dar, Ethiopia; 6 Higher Institute of Nursing Professions and Technical Health, 70000 Laayoune, Morocco

**Keywords:** Cancer, Computational biology and bioinformatics

## Abstract

Cancer is among the top causes of death, accounting for an estimated 9.6 million deaths in 2018, it appeared that approximately 500,000 people die from cancer in the United States alone annually. The SHP2 plays a major role in regulation of cell growth, proliferation, and differentiation, and functional upregulation of this enzyme is linked to oncogenesis and developmental disorders. SHP2 activity has been linked to several cancer types for which no drugs are currently available. In our study, we aimed to design peptide inhibitors against the SHP2 mutant. The crystal structure of the human Src SH2-PQpYEEIPI peptide mutant was downloaded from the protein databank. We generated several peptides from the native wild peptide using an in silico mutagenesis method, which showed that changes (P302W, Y304F, E306Q, and Q303A) might boost the peptide's affinity for binding to SHP2. Furthermore, the dynamical stability and binding affinities of the mutated peptide were confirmed using Molecular dynamics simulation and Molecular Mechanics with Generalized Born and Surface Area Solvation free energy calculations. The proposed substitution greatly enhanced the binding affinity at the residue level, according to a study that decomposed energy into its component residues. Our proposed peptide may prevent the spread of cancer by inhibiting SHP2, according to our detailed analyses of binding affinities.

## Introduction

Cancer is among the top causes of death; globally, accounting for an estimated 9.6 million deaths in 2018, it appeared that approximately 500,000 people die from cancer in the United States alone annually^1^. It has been estimated that one out of every three people will develop cancer during their lifetime. The Src homology 2 (SH2) domain-containing phosphatase-2 is expressed by the PTPN11 gene^2^, it facilitates signal transduction and is widely promoted downstream of several receptor tyrosine kinases^3^. The RAS/MAP kinase pathway cannot be fully and continuously activated without the presence of this phosphatase^4^, and among other pathways, it also affects transmission through the PI3K-AKT and JAK-STAT systems. SHP2 controls a variety of cellular functions, including migration, differentiation, proliferation, and survival. Functional overexpression of SHP2 contributes to oncogenesis and developmental problems^5^. Juvenile myelomonocytic leukaemia is primarily brought on by somatically acquired gain of function mutations in PTPN11^6^, an uncommon, aggressive, myeloproliferative illness that first appears in early infancy and has an extremely poor prognosis; no treatments are currently available. Somatic PTPN11 mutations are present in childhood myelodysplastic syndromes, acute Monocytes leukemia, and acute lymphoblastic leukemia^7^. Solid tumors include syndromes, chronic myelomonocytic leukemia, and diseases including melanoma, neuroblastoma, glioma, embryonal rhabdomyosarcoma, lung cancer, and colorectal cancer. SHP2 activity has been connected to a number of cancer types in addition to cancers brought on by PTPN11 mutations. For cancer cells driven by RTKs to survive, SHP2 is necessary^8^. It is a significant factor in both inherent and acquired resistance to some targeted cancer treatments^9^, operates as a mediator of immune checkpoint pathways^10,11^, and is also associated with Helicobacter pylori-induced gastric carcinoma^12,13^. SHP2 is implicated in two disorders that belong to a family of rare diseases known as RASopathies, in addition to its involvement in cancer^14^. In addition to its SHP2 has two Src homology 2 (SH2) domains, N-SH2 and C-SH2, followed by the catalytic PTP domain and an irregular C-terminal tail^15^. SH2 domains are protein recognition elements that engage phosphorylated tyrosine-containing protein sequences^16,17^. The connections they promote in SHP2 with RTKs, cytokine receptors, cell adhesion molecules, and scaffolding adaptors are important for SHP2. The bulk of mutations concentrate at the N-SH2/PTP interface, causing the connection between these two domains to become unstable thus activating the constitutive phosphatase^18,19^. These mutations cause an increase in responsiveness to stimulation through the association of bis-phosphorylated sequences. The RAS/MAPK signaling pathways cascade is subsequently activated in each case. The studies discussed above indicate that SHP2 is a crucial molecular target for cancer and RASopathies^20,21^. Active-site inhibitors, which lack target selectivity, have long been the main focus of SHP2-targeted drug discovery efforts^22,23^.

The study showed that just making SHP2 more active is not enough to cause disease. It's important for SHP2 to bind to other proteins, and stopping this binding could be a good way to treat the disease. The SH2 domains of SHP2 are a good target for drug design, but no drugs have been made yet^24^. Based on these factors, we explored in silico methods to create peptides that target SHP2 protein-peptide interactions rather than its catalytic activity. It is common for researchers to design peptides using computational methods^25,26^. We are currently adopting a SHP2 peptide inhibitor to conduct residue scan-based approach in order to generate more effective peptides against the oncogenic SHP2.

## Materials and methods

### Peptide modeling and Library construction

The protein structure of human Src SH2 domain Mutant complex with Inhibitory Peptide PQpYEEIPI was downloaded from (https://www.rcsb.org/structure/1KC2)^27^. The Src SH2-PQpYEEIPI complex is dimer peptide, i.e.; Chain_A containing 103 residues, while Chain_B containing eight small peptides residues including phosphorylated tyrosine residues. The retrieved structure was first checked for any missing residues and clashes. Designing peptide therapeutics or small molecule inhibitors requires a crucial understanding of the binding interface between two interacting protein partners. The same strategy was then used to implement the design of a decoy peptide targeting SHP2, using machine learning techniques in MOE^28^. The SHP2 targeting peptide was modified by substituting the least involved amino acid, PQpYEEIPI, as confirmed by alanine scanning and the eight long peptide residues were kept in the complex structure during the modification process^29^. The impact of every amino acid in the reference peptide (PQpYEEIPI) that interacts with Src SH2 protein was evaluated using an in-silico alanine scanning approach. The MOE program's alanine scanning tool was utilized to create a peptide library for the SHP2 mutant. The MOE suite's Alanine scanning module was used to compute the affinity and stability for a specific amino acid. The affinity and stability values demonstrated the proportionate change in binding energy that occurs when an amino acid is converted into alanine^30–32^. The mutation list was excluded from the native-to-alanine and native-to-native mutations to reduce computational expenses. MOE was used to scan the residues to determine how the PQpYEEIPI peptide interacted with the receptor, and stability and affinity scores were used to assess the change in binding energies resulting from the transformation of one amino acid into another. The MOE residue scan module was used in conjunction with the LowModeMD ensemble and the unary quadratic optimization (UQO) parameter. The residue scan generated a database of mutant peptides with corresponding scores. The residue scan and alanine scanning mutagenesis procedures have been previously discussed^33^.

### All atom MD simulation

The most abundant computer simulation method called MD simulation enable us plentiful information about the stability and dynamics of protein–ligand and protein–protein complexes^34,35^. The study was carried out to check the stability of Src SH2-PQpYEEIPI peptide mutant and rationally developed peptide through alanine, and residues scanning methodology. After selecting the top four peptides based on their affinity and stability, they underwent stability analysis along with the Wild-mutant complex. Amber v2022 and the ff19SB force field were utilized to conduct molecular dynamics simulations of the designed peptide mutants, which were inserted for MD simulation. Post trajectory analysis of the simulations was also performed using a GeForce RTXTM 3060 Ti^32^. All the topology files and coordinate files were prepared using tLEAP module*.* The Transferable intermolecular potential with 3 points (tip3p) water model with a box dimension of 12.0 was used to adequately solvate each system. The counter-ions, such as sodium or chloride ions, were then supplied into the solvate box by the tLEAP in order to neutralize the systems. The neutralized complexes also underwent 4000 stages of steepest descent minimization and 2000 steps of conjugate gradient minimization. Thereafter, each system was gradually heated to 298 K for the following 50 ps. With steady pressure, 100 ns of MD were executed. A Langevin thermostat was in charge of controlling the temperature (1 atm, 300 K)^36^. The Particle Mesh Ewald (PME) algorithm was used to calculate long-range interactions. For the covalent bonding, the SHAKE algorithm was employed^37^. MD simulations of five complexes were carried out using the PMEMD.cuda GPU, with the accelerated GPU pmemd used to complete the simulations for all systems. The resulting trajectories were then processed and evaluated using the CPPTRAJ module of Amber-22.

### Post trajectories analysis

Post trajectory analysis was calculated using the CPPTRAJ module of Amber-22 to study several stability parameters, RMSD (root mean square deviation), RMSF (root mean square fluctuation), hydrogen bond analysis (Hb), protein size and compactness (Rg), and DCCM (dynamic cross correlation analysis). Finally, OriginPro 9.0 software was used to construct the graphs and Visual Molecular Dynamics (VMD) to visualize the trajectory data^38^. The cut-off angle was set to 120°, and the cut-off distance was kept at 3.5 Å (between H-bond donor and acceptor atom) to calculate all hydrogen bonds (H-bonds) among the receptor and peptide mutant complex.

### MMPBSA Binding free energy calculation

The MM/PBSA method, which uses a combination of molecular mechanics and the generalized Born surface area model, was used to calculate the binding free energy of the entire complex. The topology of each system was adjusted using mbondi3 radii and 2 Å solvent probes^39,40^. The binding free energy (ΔGbind) was obtained by subtracting the protein-peptide complex energy (ΔGR + L) from the receptor energy (ΔGR) and ligand energy (ΔGL), using the equation. The binding energy for both the wild-type and mutant systems was calculated using the Python package MMPBSA.py in Amber-22^41^. The MM-PBSA study assesses a realistic prediction of the free-binding energy, interfaces between the atoms of the peptide and protein, and the subsequent impact of the solvent environment on the peptide binding affinities. All calculations were done as a percentage of the total binding energy to determine the amounts of van der Waals (vdW), electrostatic, Generalized Born (GB), and solvent-accessible (SA) energies. To calculate the free energy, we used the following equation:

$${\text{G}}_{{{\text{bind}}}} = \, \Delta {\text{G}}_{{{\text{complex}}}} - \, \left[ {\Delta {\text{G}}_{{{\text{receptor}}}} + \, \Delta {\text{G}}_{{{\text{ligand}}}} } \right].$$

The term G_bind_ in the equation above denotes total binding free energy, whereas the other terms refer to the binding free energies of the complex, protein, and ligand molecules.

### Protein clustering analysis

Principal Component Analysis (PCA), an unsupervised learning technique, was carried out to analyze the motion of molecular dynamics (MD) trajectories and better understand the functional mobility of macromolecules. The analysis focused on the protein's internal motion. To conduct the PCA analysis, we employed the cpptraj package to process the Cartesian coordinates of carbon (C) atoms for 6000 snapshots of each simulation throughout the trajectory. We generated the positional covariance matrix and eigenvector covariance matrix to investigate the dynamic behavior of each system. The diagonal transformation of the positional covariance matrix produced diagonal eigenvalues and eigenvectors, which were used to represent the PCA. The motions of each simulation were tracked by calculating and plotting PC1 and PC2 for the first and last two parameters. By free energy landscape analysis, the lowest energy stable state of macromolecules, or the free energy landscape (FEL), was captured. Deep valley margins depict the intermediate conformations, while deep valleys themselves show the lowest-energy stable state.

1 $${\text{G }} = \, - {\text{KBTlnP }}\left[ {{\text{PC1}}/{\text{PC2}}} \right] \, = \, \left[ {{\text{PC1}}/{\text{PC2}}} \right]$$

Equation 1, in which (−KB) stands for the Boltzmann constant and [PC1/PC2] for reaction coordination, P stands for the PCs' probability function, [H] for enthalpy change, [G] for total energy, and [S] for entropy.

## Result and discussion

### Retrieval and preparation of initial structure

**Figure 1:**
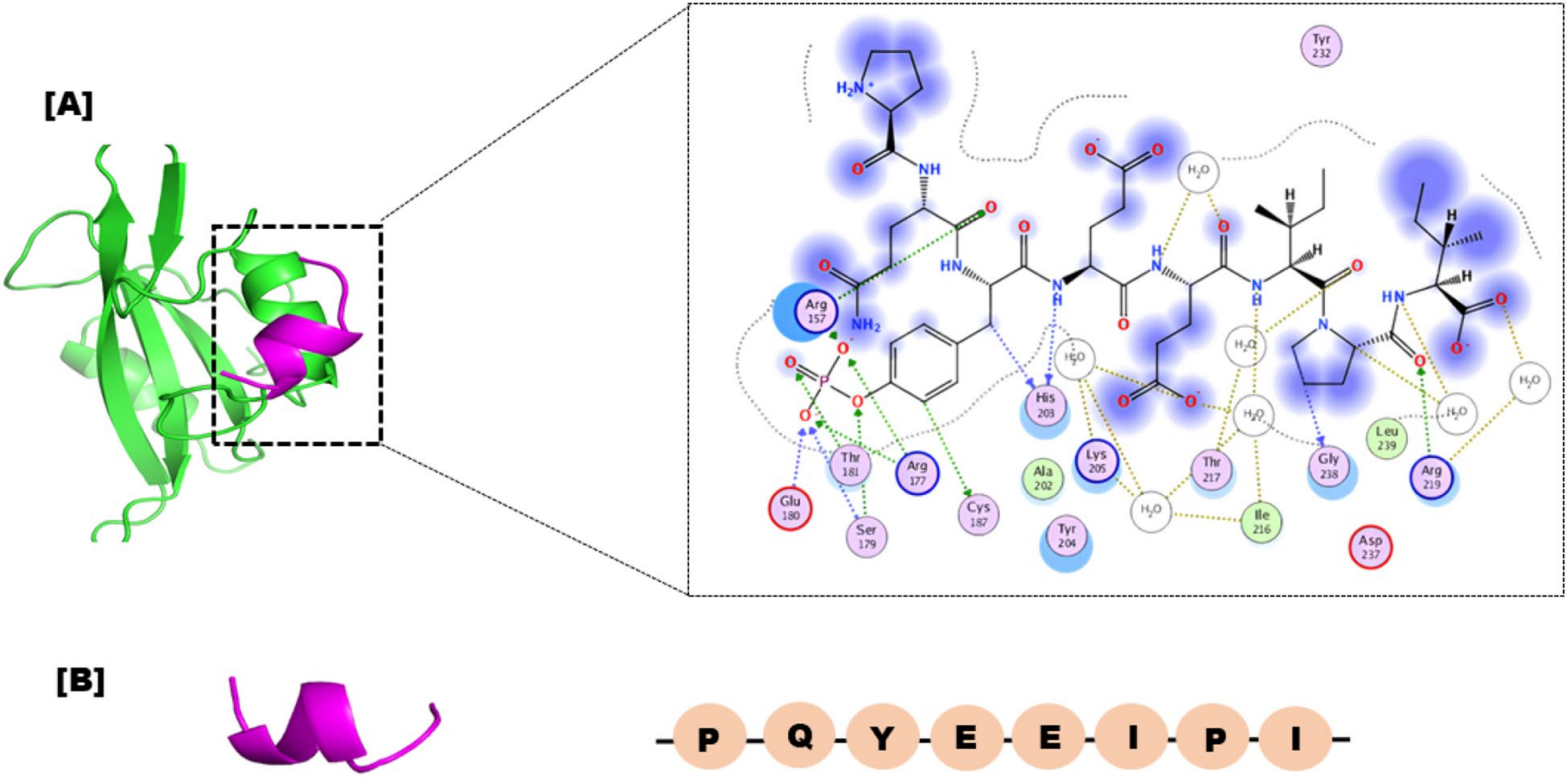
The 3D structure of Src SHP2 in green colour bound with peptide inhibitor in magentas colour. Detailed interaction of the eight residue peptide with the receptor containing residues such as Ser223, Gln227, Tyr231, Phe222, and Thr220 are shown in the right side.

The crystal structure of the SHP2 was retrieved from RCSB using the PDB number;1KC2. The protein model's protonation state was set to neutral pH using the MOE2020 software. Partial charges were then added, and the MMFF94x force field was used to minimize the model's 3D coordinates. During the minimization process, all heavy atoms were kept in position until the root mean square deviation (RMSD) gradient reached 0.1 kcal/mol. We used the London dG scoring function and the GBVI/WSA rescoring method in combination with the Triangle matcher docking algorithm to predict the binding of the peptide (PQpYEEIPI) to the SHP2 protein. After the docking stage, we utilized a force field-based scoring function for the post-docking refinement process. Figure 1 shows the structure of the SHP2 protein and the peptide of interest.

### The Mutant Structure Interface Analysis and Designing of peptide library

The human Src SH2 protein has a 103 residue structure, and it forms a complex with an eight amino acid PQpYEEIPI peptide, known as SHP2/PQpYEEIPI. The retrieved protein's PQpYEEIPI peptide mutant formed five hydrogen bonds with the SHP2 protein. It can be seen that Ser223, Gln227, Tyr231, Phe222, and Thr220 are involved in hydrogen bonding with the peptide. To determine how the PQpYEEIPI peptide functions within the SHP2 receptor, the MOE program was used to conduct residue scanning. The residue scan technique, was utilized to identify the surface residues between the peptide and the SHP2 protein. In the Wild-peptide, the residues HIS203, CYS187, GLY238, ARG157, SER179, and THR181 were chosen as site residues, and they interact strongly with the receptor protein. The other residues were demonstrated to be less important at the interface. We first used an alanine scanning technique to pinpoint the ideal replacement residue in order to increase the binding affinity of the reference peptide. The selection of promising residues for mutation can be aided by the Alanine Scan method. The Wild-peptide's nine residues were all analyzed utilizing the Alanine Scan technique. These findings suggest that only four mutants showed high negative affinity values, indicating that interactions with them were more important, whereas the rest of mutants showed extremely high positive dAffinity values, indicating that interactions with the receptor were less important. In order to find the most important residues, these strategies were implemented, determines changes in the binding affinities upon residues substitution. The alanine scanning analysis identified six residues that could potentially impact the interaction of the peptide. Substitution of these residues, namely HIS203, CYS187, GLY238, ARG157, SER179, and THR181, with other amino acids could improve the binding affinity of the peptide. The residue scan investigation involved comparing the binding energy of the wild-type residue with that of the corresponding mutant amino acid residue. The binding affinities of each of the fifteen distinct shortened amino acids used to replace each chosen residue were computed.

**Figure 2:**
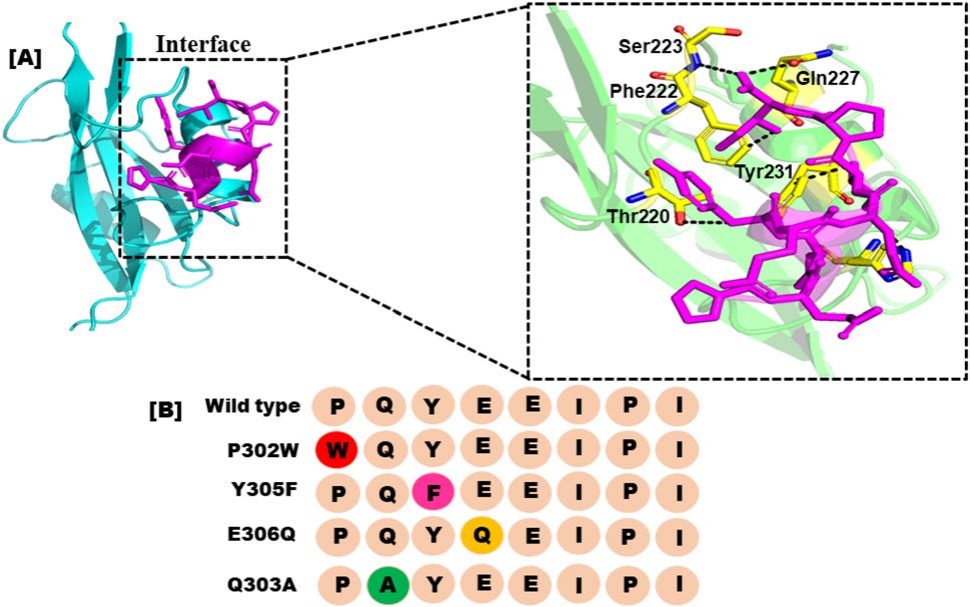
(**A**) Represents the actual peptide's and the SHP2's interface study. (**B**) Each amino acid replacement and the developed peptide library have a different color.

Four distinct peptides were created using residue scanning. These eight distinctively altered peptides were put through a permutations analysis, which produced a number of engineered residue combine with peptide inhibitors. Herein, the original sequence of peptide was altered at residues “PQpYEEIPI” to produce a library of peptide inhibitors with a length of eight amino acids new numbering start from 302 to 310. The paper demonstrated how permutation analysis was used to construct the library of peptide per-mutants using the MOE suite. The final peptide library was created using these techniques, resulting 175 peptides mutants, each peptide having one mutations (per-mutant). Multiple mutant peptides differ by more residues from the reference peptide, while one-point mutant peptides differ by one substitution and one residue. To calculate dAffinity for this residue scan analysis, the binding energies of the wild-type residue and the corresponding mutant amino acid residue were compared. The top four one-point modified peptides were chosen from a pool of 175 mutant peptides based on the Affinity and dAffinity values Table 1. With falling dAffinity levels, the peptides' stability will rise. The negative value suggested that there was a significant interaction. Figure 2 depicts the interface and the planned peptide library structure.

**Table 1:** Computationally peptide designing by replacing residues in (PQpYEEIPI).

S.NO	Peptide	Affinity	dAffinity
1	P302W	−13.1194	−1.37
2	Y305F	−12.0811	−1.86
3	E306Q	−12.284	−1.73
4	Q303A	−13.666	−0.86
WT	PQpYEEIPI	−7.433	0

### Finally, selected peptide mutant binding interactions

**Figure 3:**
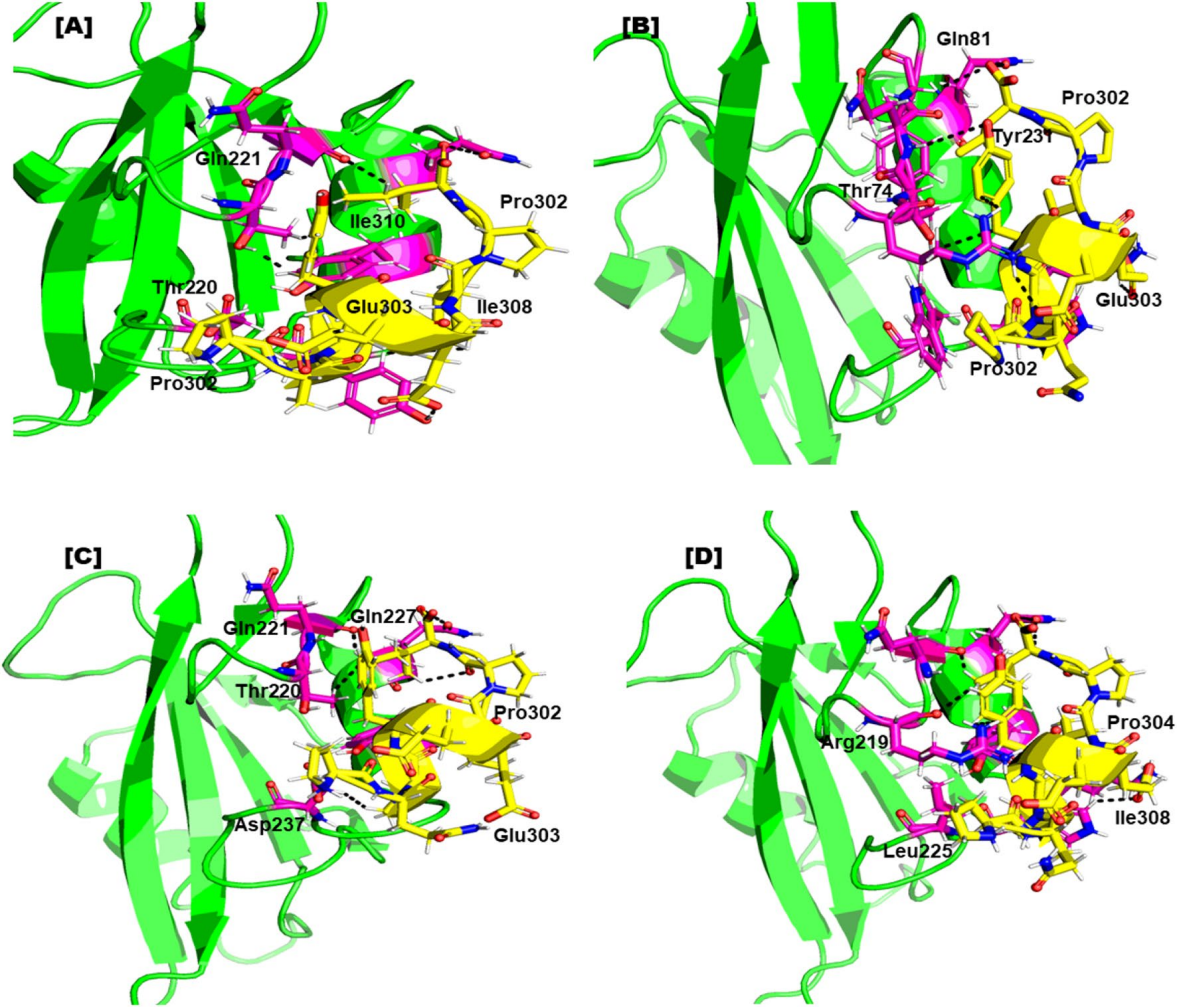
Represents the binding residues of the finally selected peptide after MD simulation (**A**); The binding mode reveals the designed peptide mutant WQPYEEIPI (P302W), formed 6 H-bonds with the active site residues of the receptor. (**B**) interaction pattern of WQPFEEIPI (Y305F) with the SHP2 resulting four H-bond. (**C**) Detailed interaction of WQPFQEIPI (E306Q) with the receptor forming five H-bond. (**D**) interaction pattern of WAPFQEIPI (Q303A) with the receptor after the simulation with total five H-bond. All hydrogen bond are represented by a solid black line.

It is well known that protein–protein docking offers crucial guidance for the generation and discovery of new pharmaceuticals. The intensity of the interactions between the receptor and peptide is measured by the S-score. Comparing the finalized mutant’s peptide to the positive control in the docking investigation, all of them showed good interactions toward the SHP2 catalytic site. In the 3D crystal structure, the PQpYEEIPI peptide is connected to the SHP2 active residues. Our investigation into hydrogen bonding, as presented in Fig. 3, revealed that all of the selected peptide variants exhibited robust and optimal hydrogen bonding networks with the SHP2 protein. When compared to the Control protein-peptide complex, each of the produced peptide/complex systems, such as, the designed peptide **E306Q**, have numerous hydrogen bonds. The outcomes of our investigation indicate that each of the produced peptides have high, exceptional SHP2 binding potential and may operate strong peptide inhibitors. To confirm the effectiveness of these peptide inhibitors in treating cancer, additional experimental research, such as SHP2 in vitro binding assays, are scheduled to be conducted in the near future.

### Analysis of the final Src SH2-PQpYEEIPI peptide mutant complexes using molecular dynamics simulation

**Figure 4:**
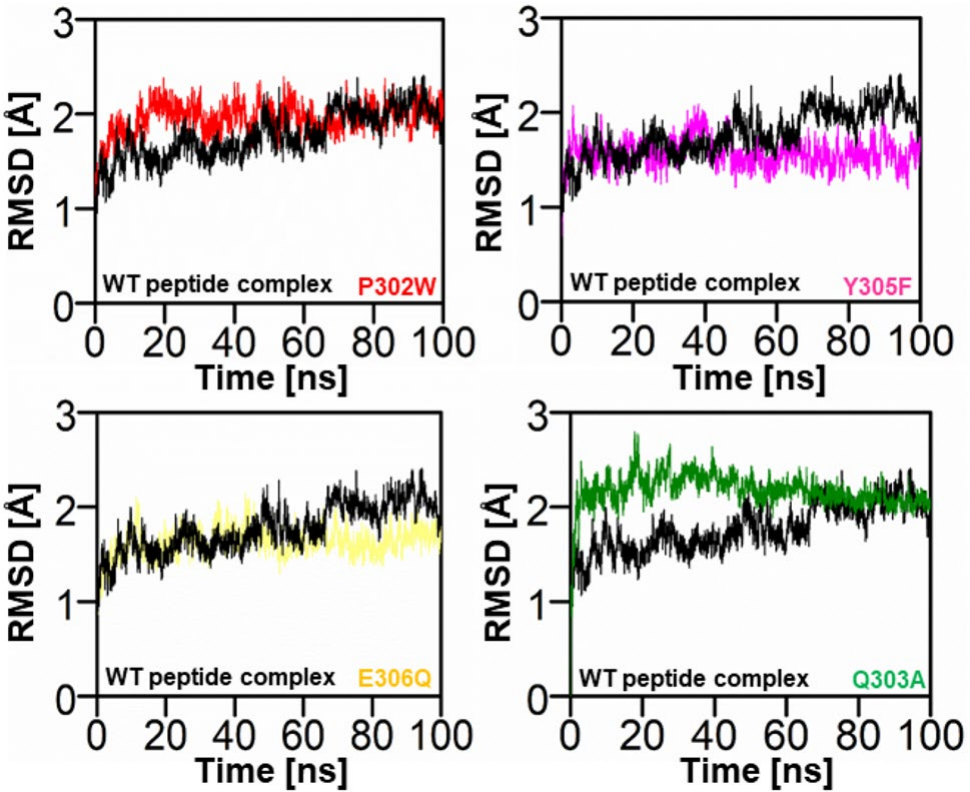
Plot of the wild and mutant peptide complexes’ root mean square deviations. The x-axis and y-axis showed the time in nanoseconds (100 ns) and RMSD in Angstroms respectively. (red) RMSD of P302W Peptide/complex, (Magentas) Y305F Peptide/protein complex, (Light Yellow) RMSD of E306Q, and (green) RMSD of Q303A respectively.

In order to determine the acquired Src SH2-PQpYEEIPI peptide mutant, a 100 ns MDS was carried out using the AMBER 22 software. The top-active four selected peptide/protein systems, and the Wild-peptide/protein complex were all subjected to MDS using the AMBER-22 programme. The four finalized Src SH2-PQpYEEIPI peptide complexes were evaluated for stability by calculating the RMSD of the backbone atoms. The stability of the system was inversely correlated with the amplitudes of the C atom fluctuation. As the RMSD variation decreases, the system becomes more stable and experiences smaller fluctuations of the C atoms^42^. The complex's stability is under-stood by the RMSD plot Fig. 4, and its structural flexibility by the RMSF plot.

An MDS equilibration could be accomplished in 100 ns using the P302W (red) system. The RMSD graph showed that the variations were more pronounced between 10 and 30 ns, with a score of 2.5 Å, and less noticeable after 40 ns, with a score of 1.9 Å. The system was fully stabilised with an RMSD of 1.9 Å after 40 to 100 ns, and the fewest fluctuations were seen. At 35 ns of MDS, the peptide/complex showed more variations and was less stable by up to 2. The system was then stabilized, dissipating minor instabilities with an RMSD of 1.5 Å, as shown in Fig. 4 (magentas). After 35 ns of MDS with an RMSD score of 1.5 Å, the Y305F dissipated instability up to 100 ns of simulations. Smaller fluctuations with an RMSD of roughly 1.6 were observed in the structural core of the complex system shortly after 80 ns of MDS (E306Q). Changes in the conformation of the SHP2 can be seen from the fluctuations in the backbone root mean square deviation (RMSD) in peptide mutant complexes. The Q303A peptide/protein complex initially displayed more fluctuation, which was recorded from 0 to 80 ns of simulation, but it was observed in the complex system's structural core shortly after 80 ns.

### Root mean square fluctuation (RMSF)

**Figure 5:**
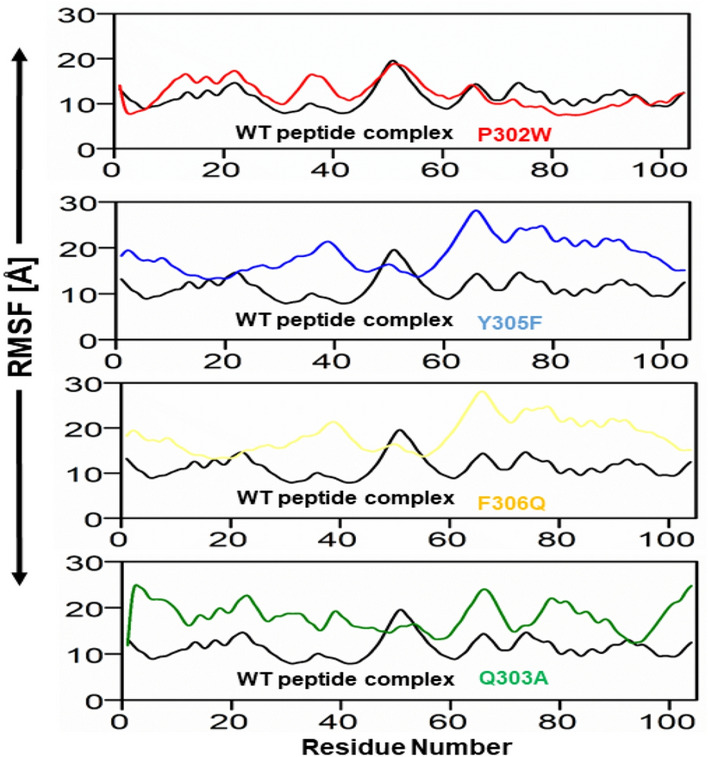
Plot of the wild and mutant peptide complexes’ root mean square deviations. The x-axis and y-axis showed the Residue number and RMSF in Angstroms respectively. (red) RMSF of P302W Peptide/complex, (Blue) Y305F Peptide/protein complex, (Light Yellow) RMSF of E306Q, and (light green) RMSF of Q303A respectively.

RMSF was used to evaluate the fluctuations of protein residues upon peptide binding Fig. 5. RMSF (root mean square fluctuation) trajectories can be used to determine the stability of each system. A plot with a great deal of motion suggests an unstable connection. A low number, or less fluctuation, on the other hand, indicates well-structured and less distorted complex regions. The amino acid level mobility in the system was evaluated by estimating the system's RMSF, it was calculated the RMSF of the backbone atoms for the SHP2 residues comprising the mutant design peptide. The stability of the peptide mutant with the biological target was confirmed by the finding that The peptide-interacting active site residues were stable and did not alter over time. Most alterations were found in distant regions from the peptide binding site, but some were observed in the flexible loop region. RMSF variations in the complexes were much lower than in the other complex, suggesting that they had lower structural mobility. The patterns produced by all systems were remarkably similar and depicted in various colors.

### Hydrogen bond analysis

**Figure 6:**
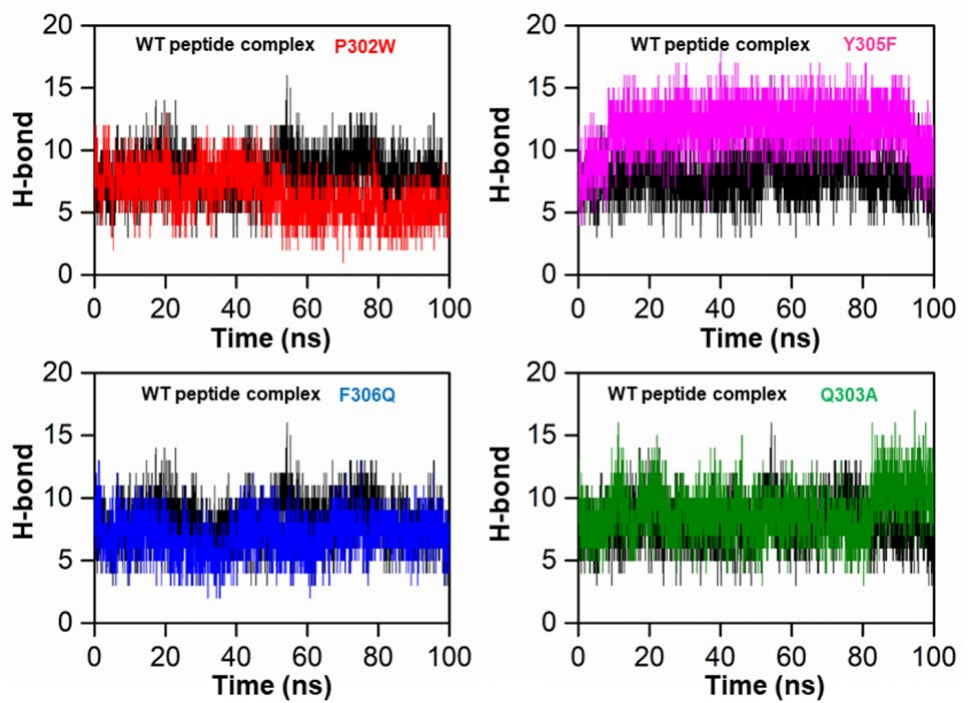
Represents the total number of hydrogen bonds in the wild peptide mutant (black) and the designed peptide protein complex, P302W (red), Y305F (cyan), F306Q (blue), and Y308R (green), respectively.

To assess the systems at the atomic level, the total number of hydrogen bonds created and the hydrogen bond was calculated in each system by using specific criteria. The criteria included a donor–acceptor separation of 0.35 nm and a hydrogen donor–acceptor angle of 30 degrees. When both conditions were met, a hydrogen bond was considered formed. Hydrogen bonding plays a critical role in maintaining the secondary structure of peptides and proteins^42,43^. The time-dependent investigation of hydrogen bonding in Fig. 6 showed that all four peptide variants recommended had strong hydrogen-bonding networks with the SHP2 protein. Each of the peptide/complex complexes produced had a significant number of hydrogen bonds compared to the wild-type complex, including the Y305F peptide/SHP2 combination Fig. 6. These findings suggest that the four peptides have a strong potential to inhibit SHP2 and could be used as effective peptide inhibitors.

### DCCM

**Figure 7:**
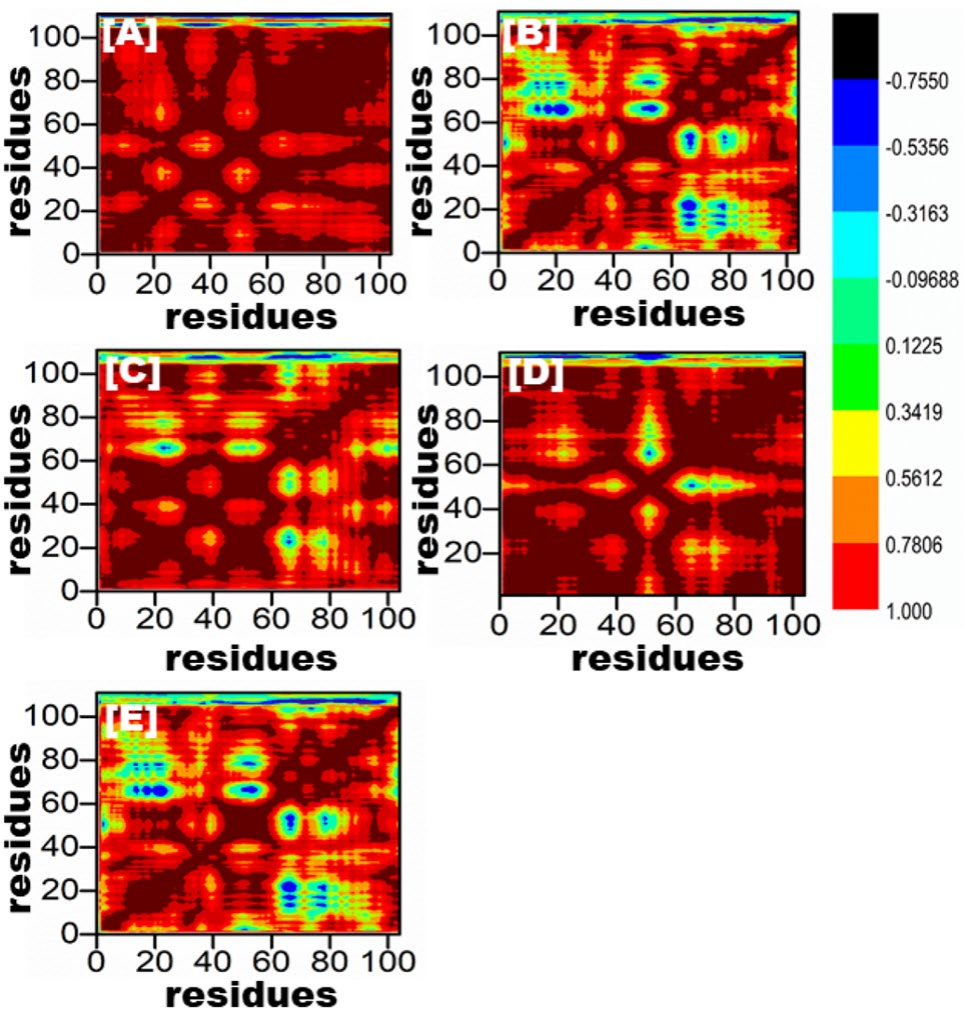
All of the mutant peptide complexes (**B**–**E**) and the wild-type are depicted in the dynamics cross-correlation matrix in (**A**). This graph displays the number of residues produced by each system during the course of a 100 ns simulation.

To explore the relationship between the motions of amino acid residues in the simulation systems, we analyzed the dynamics cross-correlation matrices (DCCM). The DCCM analysis enabled us to identify correlated or anti-correlated motions between amino acid residues in the peptide and complex systems. A black-colored region in the DCCM plot indicates a positive correlation, meaning that the amino acid residues move in the same direction, while a red-colored region indicates a negative correlation, suggesting that the residues move in opposite directions. Positive and negative correlations were represented by colors ranging from black to light blue and red to yellow, respectively, with stronger correlations indicated by deeper colors. Figure 7 shows the DCCM plot, which we used to compare the proposed peptide/complex system to the wild-type complex. Our analysis of DCCM patterns provides insights into the correlated and anti-correlated motions of amino acid residues in peptides and complexes and may have implications for the design of novel therapeutics.

### Protein structural changes analysis

**Figure 8:**
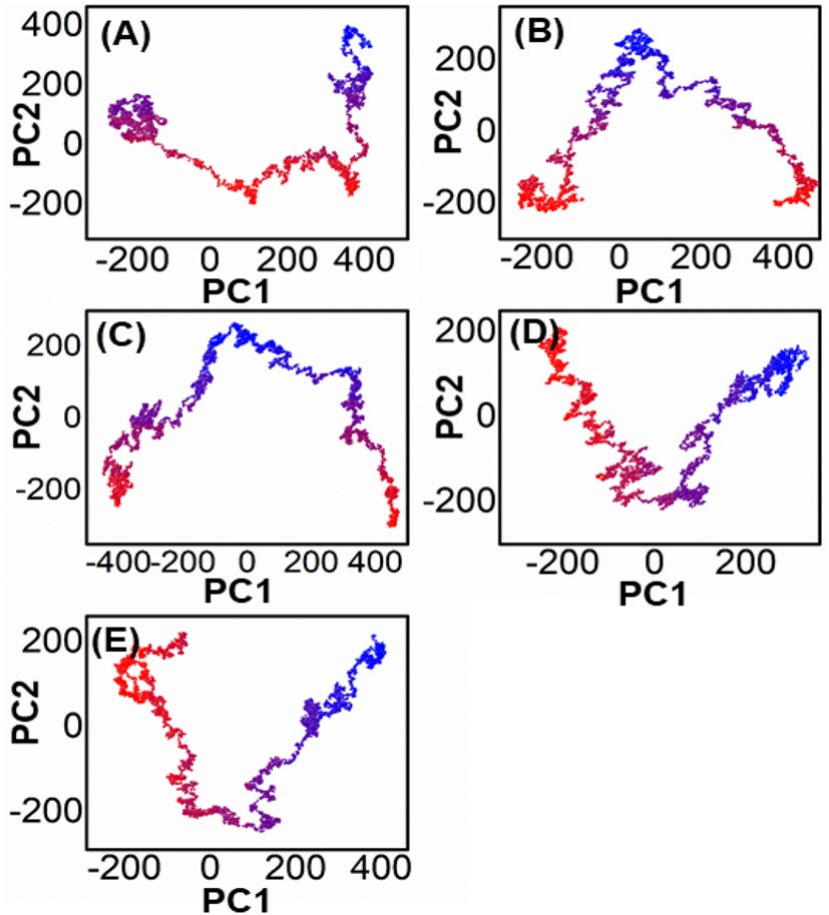
Represents the PCA analysis of all the mutated peptide complexes (**B**–**E**), and wild-type is shown in (**A**).

To identify the most prominent structural changes that the designed peptides and complexes exhibited upon binding to each system, PCA was carried out and plotted. The main objective of the 100-ns molecular dynamics (MD) simulation trajectory was to extract information about the conformational states and design of the wild-type and peptide complexes through principal component analysis (PCA). The first four eigenvectors showed significant motion in all systems, while the remaining eigenvectors exhibited local fluctuations. Figure 8 illustrates the eigenvectors and eigenvalues of the covariance matrix used to calculate the total combined motion of C-atoms in the peptides and complexes through the PCA method. The continuous red-to-blue colour transition shows the time-dependent switching between different conformations during the simulation, with each frame's alignment depicted by dots that start with red and end with blue. These results demonstrate the effectiveness of PCA analysis in elucidating the conformational dynamics of peptides and complexes, which can aid in the development of novel therapeutics. Whereas the wild-type and P302W peptide complexes exhibit more frequent leaps and continuous overlap, the Y305F, F306Q, and Y308R peptide complexes exhibit localised fluctuations. All of these findings suggest that mutations alter the complexes' internal dynamics and have a major impact on their structure.

**Figure 9:**
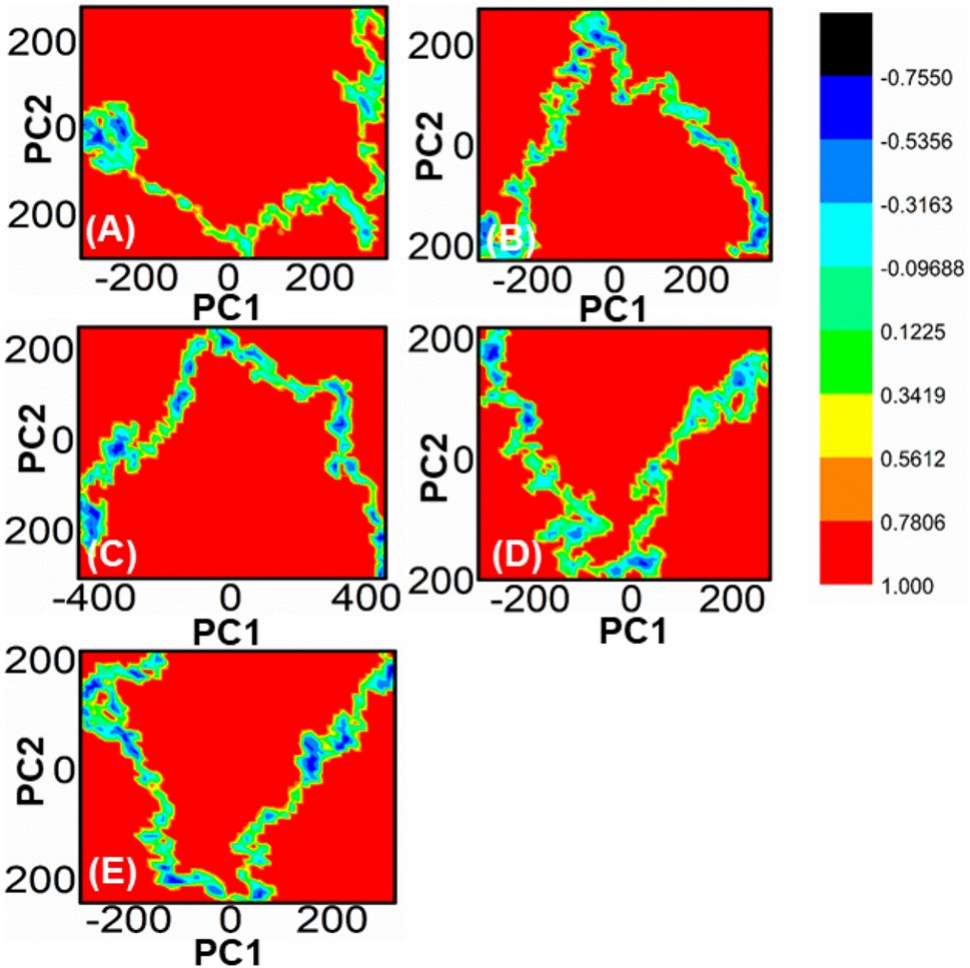
Each plot represents the free energy landscape of all the mutated peptide complexes (**B**–**E**), and wild-type is shown in (**A**).

The analysis of the free energy landscape (FEL) through principal component analysis (PCA) presents a more accurate representation of the energy and time-dependent conformational space of proteins. This computational approach distinguishes between the kinetic and thermodynamic properties of proteins, making it a valuable tool for studying their dynamics. Figure 9 illustrates the FEL plot for the first two components (PC1 and PC2), displaying the fluctuations in Gibbs free energy from the deep red to the dark blue sections that indicate the shift of the protein from a high-energy, unstable state to a low-energy, stable one over a fictitious time scale of 100 ns. The FEL analysis captures the conformational dynamics of the first two significant parts of WT proteins and complexes, and the overall results reveal that the system consistently moves towards more stable energy levels.

### Free energy calculation between Peptide/SHP2

The MM-PBSA/GBSA method is used to compute binding free energy. The molecular mechanical energies and continuous solvent fashions underpin the MMPBSA/GBSA technique. MMPBSA.py, a Python programmed, was used to determine the free energy of the wild peptide and the mutant complex. This module also looked at how to figure out the binding energies of some of the top selected peptide mutants depending on how well they fit into the binding pocket in the receptor molecules. The active peptide mutants (P302W, Y305F, E306Q, and Q303A) had an even lower MMGBSA score of −90.1851 ± 9.8727, −81.7226 ± 9.5887, −72.4711 ± 8.7426, and −77.1253 ± 9.2462 kcal/mol, respectively. Based on these results, it can be deduced that the computationally design peptide mutant that was retrieved had a free binding energy score (average ± standard error of the mean (SEM)) that was higher than the wild-type peptide mutant (−53.4198 ± 0.5471 kcal/mol).

## Conclusion remarks

Here, we used In silico mutagenesis for designing an oncogenic SHP2 peptide has shown promising results in inhibiting cancer progression. By analyzing the structural dynamics and calculating the free energy, the modified SHP2 peptide has been shown to have improved binding affinity and stability compared to the wild-type peptide. These findings highlight the potential of computational tools in designing and optimizing therapeutic peptides for cancer treatment. Further studies, including in vitro and in vivo experiments, are necessary to validate these results and explore the clinical implications of this approach.

## Data Availability

All data generated or analysed during this study are included in this published article.
